# Restricted reproductive health and infectious diseases outcomes: A call to action

**DOI:** 10.1017/ash.2022.281

**Published:** 2022-08-10

**Authors:** Pamela Bailey, Julie Ann Justo, Priya Nori

**Affiliations:** 1 Prisma Health Midlands, Columbia, South Carolina; 2 University of South Carolina School of Medicine, Columbia, South Carolina; 3 University of South Carolina College of Pharmacy, Columbia, South Carolina; 4 Division of Infectious Diseases, Department of Medicine, Montefiore Health System, Albert Einstein College of Medicine, Bronx, New York

In *Dobbs v Jackson Women’s Health Organization*, the precedents of *Roe v Wade* and *Planned Parenthood of Southeastern Pennsylvania v Casey* were overturned and a person’s right to abortion became severely restricted across the United States. In the opinion of this group of infectious disease professionals and others, the Supreme Court decision is antithetical to the Hippocratic concept of ‘do no harm’ and undermines the sanctified provider–patient relationship underpinning modern healthcare.

Legalization of abortion in the United States in 1973 dramatically reduced infection-related morbidity and mortality from unsafe abortions.^1^ The proportion of unsafe abortions is significantly higher in countries with highly restrictive abortion laws than in those with less restrictions.^2^ Moreover, until now, unsafe abortions were more commonly associated with developing countries, rather than wealthier nations, such as the United States.^2^ With severe restrictions on abortion in multiple states in the United States, healthcare providers anticipate a sharp rise in complications from unsafe abortions and unsafe pregnancies. The US Supreme Court ruling is expected to disproportionately affect people of color and those of low socioeconomic status, groups which the US healthcare system cannot afford to marginalize with further structural barriers to care. In 2020, prior to the current ruling, the maternal mortality rate for non-Hispanic Black women in the United States was 55.3 deaths per 100,000 live births—2.9 times the rate for non-Hispanic White women, a statistic that will worsen because of more restrictive abortion laws.^3^ We are now asking these women to access healthcare from an inhospitable system when complications from unsafe pregnancies or abortions arise.

As infectious disease professionals, this ruling directly affects the care of our patients. At this juncture, we must re-evaluate best practices for managing septic abortion—any abortion (spontaneous or induced) complicated by replicating bacteria in the retained placental tissue, spreading via the maternal villous space and the decidua of the endometrium into the genital tract and potentially the bloodstream.^4–6^ Across various settings, complications from septic abortion are the leading causes of abortion-related deaths, and ∼10% of maternal deaths worldwide are attributable to septic complications.^4,5,7^

Treatment of septic abortion is antibiotic therapy with prompt surgical removal or evacuation of retained products of conception. Although surgical treatment is not within our domain as infectious disease providers, ensuring appropriate antibiotic therapy is squarely our responsibility. In a 2016 Cochrane review on antibiotics for treating septic abortion, only 3 small randomized controlled trials had been conducted in >30 years prior that defined treatment options.^5^ A 2015 Cochrane review of endometritis treatment notes greater risk of failure with certain regimens exhibiting poor activity against antibiotic-resistant bacteria.^8^ This finding suggests that local epidemiology and antimicrobial resistance rates are key factors to consider when managing septic abortions. Therefore, as medical professionals, we are currently at a crossroads of multiple intersecting public health crises: loss of bodily autonomy, poor maternal outcomes, and antimicrobial resistance.

In an era of clinicians with limited experience in treating septic abortion, early recognition and treatment are critical. A “*Clostridium sordellii–*like toxic shock” (CSTS) syndrome includes rapid progression of symptoms to death, frequently within 24–48 hours of symptom onset. Progesterone and mifepristone, as well as the pH of the amniotic fluid, create an environment allowing for germination of the spores of *Clostridium* spp, and the spores then generate the toxin release that drives subsequent multiple-organ system failure.^1,4^ Without prompt recognition of this syndrome and initiation of combined surgical and antimicrobial treatment, women may succumb to sepsis.

Although CSTS is the most alarming complication, definitive pathogen diagnosis is often elusive in these cases. Enterobacterales are the most common pathogens identified, though infections are commonly polymicrobial (∼24%).^7^ The World Health Organization (WHO) recommends a combination of ampicillin plus gentamicin as empiric treatment for serious pelvic infection, or amoxicillin if not severe.^6^ This regimen of ampicillin and gentamicin is based on limited data from before 1975, which must be re-evaluated in 2022 amid this crisis. Newer or more streamlined antibiotic regimens may be more appropriate and reflective of current infectious disease practices.

From an infection prevention perspective, women who require hospitalization for complications of pregnancy or unsafe abortions will be at risk for healthcare-associated infections (HAIs), including surgical-site infections, catheter-associated bloodstream infections, and urinary tract infections. The Centers for Disease Control and Prevention (CDC) estimate that on a given day, 1 in 31 hospitalized patients develops at least 1 HAI. Unfortunately, the COVID-19 pandemic has significantly worsened HAI rates, and hospitals are unfavorably positioned to tackle new challenges in addition to existing ones (ie, an influx of patients with septic abortions admitted to hospitals already struggling with excess HAIs).^9,10^

An increase in septic abortions and pregnancy complications in the aftermath of the COVID-19 pandemic is likely to add additional strain on the fragile US healthcare system. The pandemic resulted in healthcare workers leaving the field in droves. Additionally, the modern healthcare worker has generally not provided patient care in a world where abortions could not be obtained legally and safely (*Roe* was decided on January 22, 1973). Eschenbach noted that before 1973, septic abortions were an integral part of obstetrics and gynecology residency training, but now they are rarely encountered.^4^ Limited personnel and lack of experience, combined with antiquated antimicrobial recommendations, render us ill-prepared to face this crisis.

In 2018, only 2 deaths related to legal abortions were identified by the CDC,^11^ emphasizing the safety and favorable health outcomes inherent in this procedure. As healthcare providers focused on patient outcomes from infectious diseases, we remain committed to doing no harm, practicing empathy, and creating a safe environment for patients. The US Supreme Court decision restricting the reproductive health of women renders an already difficult calling more fraught. A legal decision mired in polarized ideology and politics has invaded the sanctity of the provider–patient relationship and eroded the already fragile trust of patients cared for within the US healthcare system. In addition to restricting bodily autonomy and ensuring that certain groups of women remain in poverty, we are deeply concerned about unfavorable infectious disease outcomes inherent in a ruling that disproportionately affects marginalized and at-risk individuals who can become pregnant.

It is our civic and professional duty to advocate for reproductive rights and unrestricted access to healthcare. This includes being active in local elections, policy development, and education of our communities. In addition, we call on infectious disease professionals to conduct formal re-evaluation and research into optimal antimicrobial regimens for the management of septic abortions. It is time to contribute to the global understanding of this disease state to improve the quality of care for this vulnerable patient population.
